# A Selective Assay to Detect Chitin and Biologically Active Nano-Machineries for Chitin-Biosynthesis with Their Intrinsic Chitin-Synthase Molecules

**DOI:** 10.3390/ijms11093122

**Published:** 2010-09-07

**Authors:** Yury Herasimenka, Marta Kotasinska, Stefan Walter, Hildgund Schrempf

**Affiliations:** FB Biology/Chemistry, Applied Genetics of Microorganisms, University Osnabrück, Barbarastr. 13, D-49069 Osnabrück, Germany; E-Mails: herasimenka@gmail.com (Y.H.); marta.kotasinska@gmx.de (M.K.); stefan.walter@biologie.uni-osnabrueck.de (S.W.)

**Keywords:** chitin-assay, chitin-binding protein, chitin synthase, electron microscopy, polysaccharides

## Abstract

A new assay system for chitin has been developed. It comprises the chitin-binding protein ChbB in fusion with a His-tag as well as with a Strep-tag, the latter of which was chemically coupled to horseradish peroxidase. With the resulting complex, minimal quantities of chitin are photometrically detectable. In addition, the assay allows rapid scoring of the activity of chitin-synthases. As a result, a refined procedure for the rapid purification of yeast chitosomes (nano-machineries for chitin biosynthesis) has been established. Immuno-electronmicroscopical studies of purified chitosomes, gained from a yeast strain carrying a chitin-synthase gene fused to that for GFP (green-fluorescence protein), has led to the *in situ* localization of chitin-synthase-GFP molecules within chitosomes.

## 1. Introduction

The chitin recognition proteins (CHBs) from streptomycetes are small (18–19 kDa) secreted proteins, which either highly specifically recognize α chitin (*i.e.*, CHB1, CHB2) or are less selective, such as CHB3 targeting α and β chitin, as well as chitosan, the deacetylated chitin-derivative [1–5]. Based on the findings for streptomycetes, we identified the homologous chitin-binding protein (named ChbB) from *Bacillus amyloliquefaciens* ALKO 2718, recognizing α and β chitin, but not chitosan [6]. We obtained the corresponding protein (after overexpression of the *chb*B gene in *E. coli* in frame with six histidine codons) as a homogeneous type in larger quantities, than the *Streptomyces* CHB1-type from its natural host or also after its overexpression in *E. coli* [7,8].

Previously, we had established the use of CHB1 to target specifically only α chitin within biological samples including fungi [2]. This method is superior to the use of dyes, *i.e.*, Calcofluor [9], Congo red [10] and primulin [11], as these interact with a range of polysaccharides, including cellulose. The lectin wheat-germ agglutinin (WGA) recognizes a minimum of three consecutively linked *N-*acetylglucosamine residues within glycoproteins and within crystalline and colloidal chitins [12]; thus is less specific than chitin-binding proteins [2,4].

The application of CHB1 has recently also allowed deepening our knowledge as to chitin-biosynthesis. Having purified chitin synthase I-containing vesicles (chitosomes) [13] from the yeast *Saccharomyces cerevisiae*, we viewed by electron microscopy in the presence of the substrate UDP-*N*-acetylglucosamine, the emergence of nascent chitin-filaments, which can be targeted by the CHB1 protein. As a result, we gained new insights as to the generation of chitin in α configuration [14]. The possibility to fuse a gene in frame with codons for histidine, strepavidin or the green fluorescent protein (GFP), has opened new avenues to rapidly purify, immobilize or localize the resulting His-tag, Strep-tag and GFP-fusion proteins [15,16].

In this report, we describe a selective assay to identify chitin as well as chitin-synthesizing chitosomes. In addition, we immuno-localized by electron microscopy chitinsynthase molecules tagged with GFP within purified chitosomes.

## 2. Results and Discussion

### 2.1. Features of the chbB Fusion Genes and Their Gene Products

**Figure 1:**
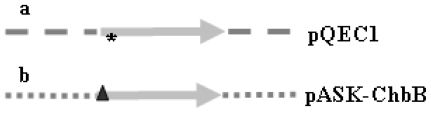
Features of the plasmids used. (**a**) The pQEC1 plasmid contains the *chb* gene (grey arrow) in frame with six codons for histidines (*) within the pQE (– – –) vector. (**b**) The pASK–CHbB plasmid; contains the *chb* gene (grey arrow) in frame with codons encoding the Strep-tag (▴) within the pASK-IBA7 vector (
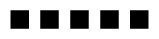
).

Previously, we had described the gene *chb*B from *Bacillus amyloliquefaciens*, encoding the chitin-binding protein ChbB (Chitin_bind_3 Superfamily, pfam03067), its fusion with six histidine-codons and its cloning, resulting in the plasmid pQEC1 (Figure 1a). The corresponding fusion protein His-tag ChbB can be obtained by affinity chromatography in larger quantities [6].

In order to establish the planned assay system (see following section), we required additionally the ChbB protein with another tag, and we selected the Strep (streptavidin)-tag. As prerequisite for this goal, we recloned the *chb*B gene in frame with codons for the Strep-tag, which are present within the pASK-IBA7 vector as outlined within the Experimental part. Selected *E. coli* XL-1 Blue transformants had the correct construct pASK-ChbB, which contained the *chb*B gene in fusion with the codons for the Strep-tag (Figure 1b).

**Figure 2:**
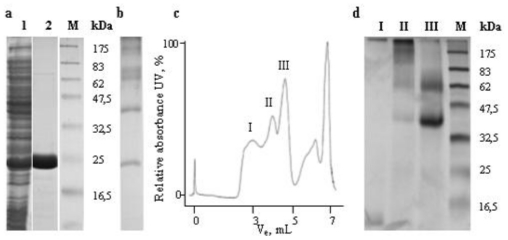
Analysis of proteins and coupling products. Proteins were separated by SDS-PAGE and stained with Commassie Brilliant Blue (a, b) or visualized after silver staining (**d**). The analysis of an aliquot of an extract (lane 1) of induced *E. coli* pASK-CHbB cells of the purified Strep-tag ChbB protein (lane 2), or reference proteins (lane M) is presented (**a**). In order to inspect the coupling products, an aliquot of the coupling reaction (see next chapter for details) among ChbB and HRP was inspected (**b**), subsequently separated by gel filtration (**c**) and a portion of the peak fractions (I, II or III) was inspected (**d**).

The fusion protein was obtained from the cytoplasm of disrupted cells (Figure 2a, lane 1) by affinity chromatography with Strep-Tactin (see Experimental part for details and legend of Figure 2 within Chapter 2.2.). We obtained larger quantities (*i.e.*, 1.5 to 2 mg per 1 L induced *E. coli* culture) of the Strep-tag ChbB fusion protein in pure form (Figure 2a, lane 2). A chitin-binding assay (see Experimental part), confirmed that the Strep-tag protein had kept its affinity to chitin.

### 2.2. Characteristics of Strep-tag ChbB Coupled with Horseradish Peroxidase

Horseradish peroxidase (HRP) is a stable hemoprotein (44 kDa) with four disulfide bridges. It is a highly stable enzyme, which has high catalytic rates of oxidoreductase-activity with many hydrogen donors to reduce hydrogen peroxide. As a result, colorimetric, fluorimetric, chemiluminescent and electrochemical assays for HRP activity are available. Additionally, after coupling of HRP with other proteins, they acquire HRP-activity [17].

In order to establish a sensitive tool to assay for the binding of Strep-tag ChbB, we aimed to conjugate it with HRP. The planned scheme included the introduction of sulfhydryl groups in Strep-tag ChbB prior to the coupling reaction. The necessity to introduce sulfhydryl-groups was dictated by the fact that the introduction of HRP (maleimide-activated) requires the free sulfhydryl groups. The sulfhydryl-maleimide coupling is a technique for the preparation of protein-to-protein conjugates. Usually, this technique has little effect on the functionality of a protein (summarized in [18]). Therefore, we introduced these groups firstly into the protected form. Their number corresponded to three moles per mole protein, and they were determined as previously described [19]. Having de-protected the sulfhydryls with hydroxylamine, the protein was mixed with HRP-maleimide in solution. This had been de-aerated, to prevent the loss of free sulfhydryls. Further analysis by PAGE, revealed a heterogeneous size-distribution of products (Figure 2b). Therefore, proteins were subjected to gel-filtration (Superdex 200 column) using FPLC; three main peaks were obtained (Figure 2c). Immunological studies (dot-blot, not shown) revealed that molecules, which reacted with *anti* ChbB antibodies, were in fractions corresponding to the peaks I, II, and III.

The proteins of peak I comprised one protein-portion of about 10 kDa, which was likely one degradation product. The second most abundant product had a molecular weight considerably larger than 175 kDa, and thus must comprise Strep-tag ChbB adducts with at least four to five molecules of HRP (Figure 2d, lane I).

Within peak II (Figure 2d, lane II) two smaller proteins, about 43–44 kDa and about 62 kDa, corresponding to non-coupled HRP and to one 1:1 adduct of Strep-tag ChbB with HRP, were found. In addition, proteins ranging from about 100 to 175 kDa and higher values were present. They were deduced to correspond to Strep-tag ChbB, being coupled with three to at least five HRP molecules. Within the peak III, non-reacted HRP (about 44 kDa) and a protein (62 kDa) corresponding to one 1:1 adduct of Strep-tag ChbB with HRP were found. Only very small quantities of higher sized adduct were present. Our data are in accordance with those obtained for coupling of IgG (immunoglobulin G) with HRP. Here, products were also poly-disperse and followed the Poisson distribution. Thus, the degree of substitution ranged from 1.5 to 5.4 and varied according to the ratio of the proteins [18]. We performed the chitin binding test using the aliquots of the proteins from the peak-fractions. The values of absorbance were 650 units (HRP-maleimide, control), 8200 units (peak I), 25490 units (peak II), and 20670 units (peak III). Highest peroxidizing activity, and hence highest level of Strep-tag ChbB-HRP, was present within the peak II-fractions. The slightly reduced peroxidizing activity within the peak III-fractions correlated with the ratio of the proteins (Figure 2d, lane III and previous paragraph) containing a higher quantity of the mono-substituted Strep-tag ChbB-HRP.

Considering the concentrations of the proteins in the peak II and III, and taking into account the presence of a huge quantity of uncoupled HRP in the peak III, we decided to take the fraction corresponding to peak II of the chromatogram (now named ChbB-HRP) for the following experiments.

Further assaying showed that Strep-tag ChbB-HRP bound most efficiently to chitin, but only little to chitosan, and not to microcrystalline cellulose and xylan (Table 1). Therefore, the designed HRP adduct, was suitable for the further studies (see next chapter).

### 2.3. His-tag ChbB and Strep-tag ChbB Are Components of a Sensitive Chitin Synthase Assay

Traditionally, chitin synthase activity has been tested using radioactively labeled substrates [20]. The lectin, wheat germ agglutinin (WGA), recognizes three consecutively linked *N*-acetylglucosamine residues including glycoproteins carrying such residues, but also chito-oligomers and high-molecular weight chitin [12]. Therefore, WGA (*i.e.*, labeled with dyes or gold-particles) can serve to monitor chitin in biological samples, if they are accompanied by proper controls with a chitinase, degrading chitin.

Later, wheat-germ agglutinin (WGA) served to establish a non-radioactive, high-throughput assay to detect chitin [21]. For this, the wells of a microtiter plate were covered with BSA (bovine serum albumin), then with the WGA-solution, and finally, a suspension containing commercial chitin, or chitin-fibers, being synthesized. Afterwards, commercially available WGA-coupled to horseradish peroxidase was added. The addition of HRP-substrate, allowed monitoring HRP activity. We had used this assay to score for the presence of chitin-synthesizing chitosomes during their isolation procedure from the yeast-cytoplasm, but due to the presence of glycoproteins, the background was relatively high.

**Figure 3:**
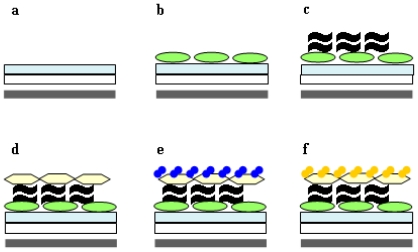
Characteristics of the chitin-detecting system. A magnet (grey bar) was placed beneath a well (white bar) of a microtiter plate and NiNTA agarose (light bluish bar) was added (**a**). A suspension of a His-tag ChbB protein (green ovals) was pipetted (**b**). The suspension with the chitin-containing sample (black wavy lines) was added (**c**). The addition of the solution (**d**) with the Strep-tag ChbB-HRP (light yellowish) and the substrates (blue dots, **e**) followed by H_2_SO_4_ (yellow dots) terminated the reaction (**f**).

Hence, we aimed to develop a highly specific test for the presence of chitin. Previously, we had shown [2,6,14] that the bacterial binding proteins CHB1 and ChbB can serve to identify either α chitin or respectively, α and β chitin (for details see Introduction). The His-tag ChbB protein (in contrast to His-tag CHB1 protein) can also be gained in larger quantities without aggregation from *E. coli.* In the course of pre-studies, we had shown that the His-tag ChbB protein can be kept immobilized on magnetic beads covered by NiNTA on the bottom of a microtiter plate in the presence of a magnet. Therefore, it was possible to replace the unselective BSA-layer of the previous test [21], by the specific chitin-binding protein His-tag ChbB (Figures 3a, b), which is fixed to the bottom of a microtiter-plate, as long as the magnet is present. Onto this layer, a suspension containing crab-chitin (as pre-test to establish the system, see Experimental part), was added. Later, crab-chitin was replaced by fraction(s) to be tested for freshly synthesized chitin (via chitosomes, see the following Chapter 2.4). Due its high selectivity [6,14], only chitin, but neither glycoproteins, nor low molecular-weight chito-oligomers bind. Hence, this layer is a superior substitute of WGA ([21], and previous paragraph). Subsequently, the chitin-containing sample under inspection (Figure 3c) was overlaid with the chitin-binding protein ChbB, which contains a Strep-tag in place of a His-tag. As a result, the Strep-tag ChbB (Figure 3d) will not bind to the NiNTA, but only to the sample, if it contains indeed chitin. In order to monitor the binding rapidly, Strep-tag-ChbB was coupled to HRP (see 2.2 and Table 1).

**Figure 4:**
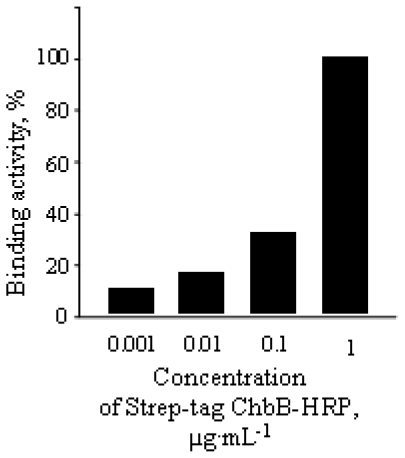
Determination of chitin-binding activity of the Strep-tag ChbB-HRP. 100 μL of a chitin-containing solution (10 mg·mL^−1^) was added to each of the four vials. Subsequently, a different concentration of the Strep-tag ChbB-HRP was applied to each vial. Then, they were washed five-times with H_2_O, subsequently the HRP substrate and 5 min later, H_2_SO_4_ was added. The solutions were centrifuged, pipetted out to the multiwell plate and the absorbance at 450 nm was measured. The binding activity for Strep-tag ChbB-HRP at the concentration 1 μg·mL^−1^ was set as 100%.

The use of crab-chitin (per well 100 μL of a chitin-containing solution/10 mg·mL^−1^) together with subsequent addition of increasing quantities (0.001 μg·mL^−1^ to 1 μg·mL^−1^ of the Strep-tag ChbB-HRP to each well, and the application of the commonly used HRP-substrate (see Experimental part), allowed us to photometrically quantify the chitin-trapped Strep-tag-ChbB-HRP (Figure 4, Figures 3e, f). To routinely evaluate assays (see next Chapter 2.4), we selected the concentration of 1 μg·mL^−1^ of the Strep-tag ChbB-HRP conjugate, as the chitosome-containing fractions were known to vary, and lead to only small quantities of chitin-fibers [14].

### 2.4. Application of the Chitin-assay to Refine the Isolation of Chitosomes

Based on previous studies, it is known that chitosomes from the yeast *Saccharomyces cerevisiae* wild type (WT) can be purified based on their sedimentation and flotation properties [20]. With this procedure, we analyzed the S. *cerevisiae* mutant strain, lacking the gene for the chitin synthase III, and succeeded for the first time to demonstrate the presence of chitin-synthase I activity within their purified chitosomes [14].

As a control, we gained chitosomes firstly according to the previously established isolation-procedure [14,20] by scoring for their active chitin-synthase within microtiter wells by applying the refined assay (see previous chapter and Figure 3).

**Figure 5:**
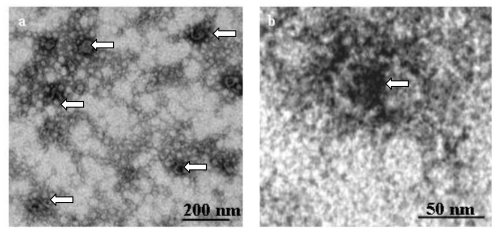
Analysis of the efficiency of the flotation gradient. Portions of fractions were assayed for the synthesis of chitin. The fraction with highest chitin-synthesizing activity was inspected by electron microscopy at two different magnifications (**a**, **b**). Some chitosomes are labeled by white arrows.

**Figure 6:**
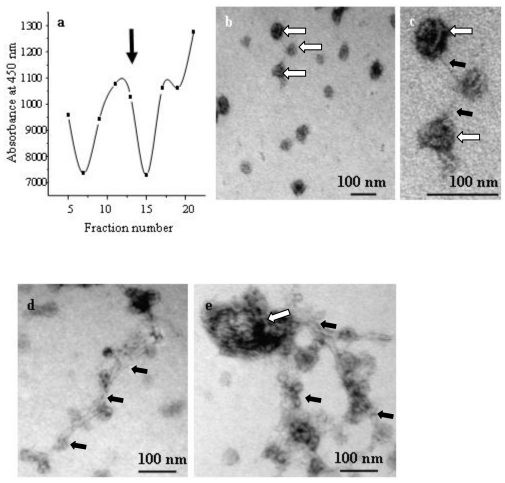
Analysis of the sedimentation gradient. After fractioning (bottom to top, and reverse numbering), a portion of each second fraction was assayed for the presence of chitin-synthase activity with the assay documented in Figure 3. The sample (fraction 13 arrow) in (**a**) contained highly pure chitosomes, which were visualized by electron microscopy at two different magnifications (**b**, **c**). Having incubated the chitosomes (c) with substrates, fibers (**d**, **e**) were generated as a result of chitin synthase activity. Chitosomes in b, which are enlarged in c, and the chitosome in e, are labeled by white arrows. Chitin-fibers are marked by black arrows in c, d, and e.

The highest chitin-synthesizing activity was present in those fractions which had a buoyant density of 1.15–1.16 g·cm^−3^. The analysis by electron microscopy showed that size and appearance of the chitosomes corresponded to those gained previously, and they produced chitin-fibers upon addition of the required substrate. Together, the data showed that the newly established assay-system allowed the detection of active chitosomes very reliably and speedily (for details see next chapter and Figures 5 and 6).

In the course of further studies, we altered several parameters including volume of tubes, rotor speed, and time for centrifugation, as well as different order of sedimentation and flotation. As a result, we found that it is best, to keep the procedure for gaining a cleared cytoplasm-extract as reported earlier (see [14] and Experimental part). However, a large improvement occurred when the cleared cytoplasm-extract was immediately placed into an empty tube, covered with the sucrose gradient and subjected to floatation in an ultracentrifuge (Figures 5a, b).

Thus, in contrast to the earlier use of sedimentation as a first step, we could apply a 10–20 fold larger sample-volume in one tube. In addition, inspection by the established test as well as by electron microscopy revealed that chitosome-containing fractions 33 to 37 % sucrose (fraction 13 in Figure 6a) had a higher purity compared to the conventional protocol, as they were already devoid of other cellular organelles. Therefore, we subjected these chitosomes-containing fractions to velocity sedimentation as the second step (see Experimental part). As controlled by the established enzymatic test and electron microscopy, the chitosomes had a high purity and intactness (Figures 6b, c). During the second centrifugation (Figure 6a) mainly individual chitosomes, (which we were interested to study), were separated (fraction 13) from those that were associated with too high (fraction 17 and 21) or low (fraction 5 and 9) amounts of membranous or fibrilliar-appearing structures (not shown). Using the procedure described above, we were also successful to gain chitosomes from *S. cerevisiae* wild-type (not shown).

In order to localize chitin synthase within chitosomes, we isolated chitosomes from the *S. cerevisiae* mutant YBR023C-GFP having the chromosomally inserted fusion gene CHS3-*gfp*. The resulting chitinsynthase molecules carry one GFP-tag at the *C*-terminus. Inspection of chitosome-containing samples by fluorescence-microscopy (data not shown) revealed that the GFP-based fluorescence was too low to be detectable.

**Figure 7:**
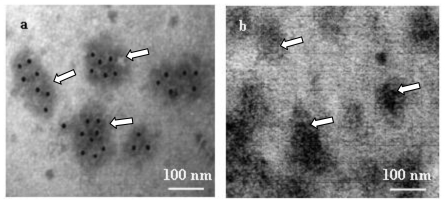
Localization of chitin-synthases within chitosomes. Chitosomes were isolated from *S. cerevisiae* containing a fusion of the *CHS3* gene with the *gfp* gene treated with primary *anti* GFP antibodies, then with secondary gold-labeled antibodies and inspected (**a**). The control (**b**) was done in the same fashion except that the incubation with the primary antibodies was left out. Some chitosomes are labeled by a white arrow (a, b). Bound secondary antibodies are visible as evenly round, electron dense particles within the chitosomes (a).

It was found to be most appropriate to incubate chitosomes directly with *anti* GFP primary antibodies, which can be raised easily against GFP (green fluorescence protein, see Introduction and [16]), and which are available commercially. The subsequent treatment was done with gold-labeled secondary antibodies (see Experimental part). Six to eight labels were found within individual chitosomes (Figure 7). Based on these results, it can be concluded that we detected the GFP-tag per molecule of the fusion-protein. As one CHS3-fusion comprises one GFP-tag, the number of gold-labels must correlate to the number of CHS3-molecules. This is the first report to evaluate the number of CHS3-molecules within a chitosome.

As strains containing *gfp-CHSI or gfp-CHS2* cannot be purchased, we did not perform the immuno-localization of the *S. cerevisiae* chitin synthases of type I and II.

## 3. Experimental Section

### 3.1. Strains, Plasmids, Growth Conditions

The *Escherichia coli* strain XL-1 Blue was from Stratagene. *E. coli* XL-1 Blue containing the pQE16-based construct pQEC1 carrying the *chb*B gene, was described earlier [6]. The *E. coli* plasmid pASK-IBA7 including codons for a Strep-tag, was from IBA GmbH. *Saccharomyces cerevisiae* wild type (WT) or the mutant 4BR023, lacking the chitin-synthase III gene (CHS3), was from a strain collection (Entian, Frankfurt). *S. cerevisiae* mutant YBR023C-GFP *c*arrying chromosomally inserted *gfp*-fusion CHS3-*gfp* was from Invitrogen, which encodes the chitin synthase 3 with a GFP portion at its C-terminal end. *E. coli* strains containing plasmids were cultivated at 37 °C in Luria Bertani medium (LB, containing 10 g of trypton, 5 g yeast extract, and 1 g NaCl per L of water, pH 7.5 [22]). If necessary, *E. coli* strains were grown on a solid LB medium containing 1.4% agar. *S. cerevisiae* strains were grown at 30 °C on a yeast malt extract medium (YM, comprising 4 g of yeast extract, 10 g of malt extract, and 4 g of glucose per L of water, pH 6.5).

### 3.2. Re-cloning of the chbB Gene and Its Analysis

The plasmid pQEC1, carrying the *chb*B gene in frame with six histidine codons [6], was isolated using the QIAprep^®^Spin Miniprep Kit (Qiagen) according to the supplied instructions. The *chb*B gene was amplified by PCR with the *Taq* polymerase (New England Biolabs) using the primer A (GACAGGATCCCACGGGTATATAAAAGAG, including a *Bam*HI recognition site) and the primer B (CGTTAAGCTTTTATTTTGTGAGGTTTAC, including a *Hind*III site). The PCR-product obtained was loaded onto an agarose gel (0.8%) in TBE buffer (0.89 M boric acid, 25 mM Na_2_EDTA in 0.89 M Tris-HCl, pH 8.0), excised and purified with the MinElute Gel Extraction kit (Qiagen). After cleavage with *Bam*HI and *Hin*dIII, the fragment was ligated with *Bam*HI/*Hin*dIII-digested pASK-IBA7. The ligation mixture was transformed into *E. coli* XL-1 Blue by electroporation. The resulting ampicillin-resistant colonies were inspected as to the presence of the designed construct pASK-ChbB using restriction enzymes. Then, DNA-sequencing was performed using the ready reaction mix and the ABI PRISM equipment (PE. Biosystems) by U.Coja (Department of Botany, University Osanbrück). Sequence analysis done with the Clone Manager Suite 7 software.

### 3.3. Purification of the Strep-tagged ChbB Protein

The *E. coli* XL-1 Blue strain containing the plasmid pASK-ChbB was grown at 37 °C in LB medium supplemented with ampicillin (100 μg·mL^−1^). The synthesis of the Strep-tag ChbB fusion protein was induced by addition of anhydrotetracycline (0.2 μg·mL^−1^) during the logarithmic phase (OD_600_ = 0.5–0.6), and the cultivation continued for 2 h. Cells were collected by centrifugation, and suspended in washing buffer (100 mM Tris-HCl, 150 mM NaCl, 1 mM EDTA, pH 8.0). After disruption of the cells by sonication (5 × 10 s, with intervals 10 s. Branson sonifier 250) and subsequent centrifugation, the gained cytoplasmic extract was mixed with 500 μL of Strep-Tactin (IBA GmbH, Germany) and incubated overnight at 4 °C. Having placed the mixture into a column, washing with five column-volumes of washing buffer followed. Then, the Strep-tag ChbB protein was released with elution buffer (100 mM Tris-HCl, 150 mM NaCl, 1 mM EDTA, 2.5 mM desthiobiotin, pH 8.0).

### 3.4. Production and Purification of the His-tag ChbB Protein

The His-tag ChbB fusion protein was produced from *E. coli* XL-1 Blue with the plasmid pQEC1 as described earlier [6].

### 3.5. Coupling of Strep-tag ChbB to the Horse Radish Peroxidase (HRP), Purification, and Identification of the Conjugate

The coupling reaction of the Strep-tag ChbB protein to horse radish peroxidase (HRP, [17]) was done by introducing sulfhydryl groups according to an earlier established method [18] with slight modifications, followed by the coupling itself, applying instructions from the supplier of maleimide-activated HRP (Sigma). Therefore, a solution comprising purified Strep-tag ChbB (see the previous chapter) was dialysed against 50 mM sodium-potassium phosphate buffer, pH 7.5, containing 1 mM EDTA (Buffer D) at 4 °C. *N*-hydroxysuccinimide ester of *S*-acetylthioacetic acid (Sigma) was dissolved shortly prior to use in DMFA (30 mg·mL^−1^), and 10 μL of this mixture were added to each mL of protein-containing solution. After reacting for 30 min, dialysis occurred against Buffer D at 4 ·C. To each mL of protein-solution 100 μL of 0.5 M hydroxylamine (in 50 mM sodium phosphate, 25 mM EDTA, pH 7.5) were added. The mixture was kept at room temperature for 2 h, and then, dialysis (against 0.15 M NaCl, 0.1 M sodium phosphate, pH 7.0, Buffer R) followed. The resulting solution with the Strep-tag ChbB protein (0.27 mg·mL^−1^), which had the introduced SH groups, was de-aerated with nitrogen. The protein containing solution was mixed with maleimide-activated HRP (in de-aerated Buffer R) to achieve a 1:1 molar ratio, and kept for 3 h at room temperature with constant mixing. The reaction was terminated by addition of β-mercaptoethanol (final concentration 1.5 mM), and was stirred for 15 min. Then dialysis was done against 50 mM potassium phosphate buffer, pH 6.5 (CHS Buffer) and used for the following experiments.

The formation of the expected product was controlled on a polyacrylamide gel electrophoresis using a 12.5% gel. If the reaction had been satisfactory, the Strep-tag ChbB-HRP conjugate was purified with an FPLC system using the Superdex 200 packed column (Pharmacia Biotech). As a liquid phase, de-aerated 50 mM potassium phosphate buffer, pH 6.5, was applied at flow rate 0.5 mL·min^−1^. The purified adduct was named Strep-tag ChbB-HRP.

To identify the complex in the course of the purification procedure, small portions of the fractions were analyzed by dot-blot analysis. For this, a Fluorotrans membrane (0.2 μm, Pall Corporation) was washed for 1 min in methanol, then, for 10 min in PBS buffer (140 mM NaCl, 2.5 mM KCl, 1.5 mM KH_2_PO_4_, 8 mM Na_2_HPO_4_, pH 7.3). A 5 μL aliquot of each selected peak-fraction was spotted onto the membrane, and subsequently, this was blocked in 5% skim milk powder containing PBS buffer for 1 h. Serum containing a*nti* ChbB antibodies [6] was diluted 1:5000 in PBS and incubated for 2 h with the membrane. After washing 3 × 10 min in PBS, incubation continued for 2 h with *anti* rabbit antibodies conjugated with alkaline phosphatase (Sigma) diluted 1:5000 in PBS.

Then, the membrane was washed twice with PBS and once in substrate buffer (25 mM Tris-HCl, pH 8.3). It was subsequently stained with naphtol-AS-E-phosphate (4 mg·400 μL^−1^ DMSO)/Fast violet B salt (20 mL, 1 mg·mL^−1^ in substrate buffer) in darkness until the color appeared. Moreover, the fractions were tested for a binding capacity to the crab chitin (see following chapters). For this purpose, they were diluted before test with CHS Buffer to a protein-concentration of 1 μg·mL^−1^.

### 3.6. Determination of Protein Concentration and the Quantity of Sulfhydryl Groups

Protein concentration was determined as described earlier [23]. The incorporation of sulfhydryl groups was estimated using Ellman’s reagent [19].

### 3.7. Binding Test and Determination of the Sensitivity

The ChbB-HRP conjugate (diluted to 1 μg·mL^−1^ in 50 mM Tris-HCl, pH 7.5 containing 1% BSA) was tested as to its binding activity [6] for the ground crab chitin (Sigma), chitosan (Sigma), xylan (Sigma) and microcrystalline cellulose (Sigma); 1 mg of each of these compounds had been suspended in 100 μL CHS buffer. After incubation for 1 h at room temperature with constant mixing, the Eppendorf tubes were centrifuged, and the pellet was washed 5 times with water. The identification of the bound ChbB-HRP conjugate was done as described [21] using as substrate a mixture of 3,3′,5,5′-tetramethylbenzidine (TMB) and H_2_O_2_ (0.8 mM TMB in DMSO, 2.15 mM H_2_O_2_ in 100 mM citrate/acetate buffer, pH 3.7). The absorbance of the colored reaction product was measured using a Tecan Sunrise™ microplate reader at 450 nm. HRP-maleimide served as a reference. The sensitivity of the ChbB-HRP was tested with ground crab chitin (2 mg) by varying the concentration of the ChbB-HRP complex from 0.001 μg·mL^−1^ to 1 μg·mL^−1^.

### 3.8. Isolation of Chitosomes

The *S. cerevisiae* strain under study (WT, 4BR023, YBR023C-GFP having CHS3-*gfp)* was grown (5 L YM-medium) until OD_600_ = 0.8–0.9 was reached. Cells were suspended, disrupted (Ribi press) and cell debris was removed as described [14]. The supernatant was subjected to ultra-centrifugation as described [14,20]. The pellet was discarded, the supernatant was filtered through 0.22 μm Millipore filter, and adjusted to a concentration of 50% sucrose in CHS buffer containing 10 mM MgCl_2_ (CHSMg buffer). The resulting 20 mL were placed into an empty (32 mL) centrifuge tube, then, 1 mL of 48% in CHSMg buffer was carefully added on top, and it was covered with 10 mL of a sucrose gradient (20–46%) in the same buffer. After ultra-centrifugation (60000 rpm, 4 °C, 16 h) using a Ti70 rotor (Beckmann), the gradient was fractionated in 0.5 mL portions. Those with the buoyant density of 1.15–1.16 g·cm^−3^ [14], which corresponded to 35–37% sucrose, were incubated with the substrate UDP-*N*-acetylglucosamine [14], and tested for resulting chitin using the ChbB-HRP conjugate (see previous chapter). Chitin-synthesizing fractions were diluted to the sucrose-concentration of 9%, and loaded onto the 10 mL sucrose gradient 12.5–65%. After ultra-centrifugation (55000 rpm, 4 °C, 3.5 h), using a TFT65.13 rotor and Centrikon T-1065 centrifuge (Kontron Instruments), the gradient was fractionated in 0.5 mL portions. Each second fraction was monitored using the ChbB-HRP conjugate (see below), and subsequently by electron microscopy.

### 3.9. Well-assay for Chitin Synthase Activity of Chitosomes, and Detection of Chitin Fibers

50 μL of the fraction was supplemented with 50 μL of the reaction mixture (GlcNAc 80 mM, UDP-GlcNAc 4 mM, MgCl_2_ 10 mM in CHS buffer), and incubated for 10 min with 10 μL of trypsin (2 mg·mL^−1^ in 50 mM Tris-HCl, pH 7.5) at 30 °C. Afterwards, the action of trypsin was blocked with 10 μL of SoyBean trypsin inhibitor (3 mg·mL^−1^ in 50 mM Tris-HCl, pH 7.5). The reaction continued for an additional 16 h at 30 °C. All operations were performed in the standard 96-well plate.

To test for the presence of synthesized chitin, 3 μL of the ChbB His-tag protein (1 μg·mL^−1^) were added to each well, and was incubated for 30 min together with 10 μL Ni-NTA magnetic beads (Qiagen), which had been pre-washed with CHS buffer. While the beads were held on a magnet, two washes were done with CHS buffer. Afterwards they were resuspended in 13 μL CHS buffer, and transferred to the well containing the sample, to be inspected for chitin-synthase activity (see above). After incubation for 1 h at room temperature with shaking, the well was washed twice with CHS buffer, while the 96-well plate was kept on the magnetic device. A portion of 100 μL ChbB-HRP (1 μg·mL^−1^ in CHS buffer) was added to each well, and incubated with shaking at room temperature for 1 h. The plate was washed five times with distilled water. To each well, 100 μL of the freshly made HRP-substrate (0.8 mM 3,3′,5,5′-tetramethylbenzidine in DMSO, 2.15 mM H_2_O_2_ in 100 mM citrate/acetate buffer, pH 3.7) was added, and incubated with shaking for 5 min. The addition of 100 μL 0.5 M H_2_SO_4_ terminated the reaction. The developed yellow color was measured using the Tecan Sunrise microplate absorbance reader (Tecan, Austria) at 450 nm.

### 3.10. Electron Microscopy and Immuno-localization

Electron microscopy was done as described [14], except that contrasting was done with 3% solution of phosphotungstic acid as outlined earlier [24]. Analyses were done with the EM 902A microscope (Zeiss).

For immunological studies, the samples were prepared as follows. Onto each grid, which had been pre-incubated with 2% BSA for 1 h, a drop of the sample was placed. The excess of the liquid was removed with the filter paper. After incubation for 30 min at RT, each grid was covered with a droplet of *anti* GFP antibodies (produced in rabbit, Sigma), which had been diluted 1:100 in PBS containing 2% BSA and incubated overnight. After six washes with 2% BSA in PBS, the grids were incubated for 4 h with diluted 1:100 *anti* rabbit antibodies, which had been marked with colloidal gold (10 nm). After three washes with PBS, the grids were contrasted with 3% phosphotungstic acid and analyzed using the EM 902A microscope (Zeiss).

## 4. Conclusions

The presented test system relies on the chitin-binding protein ChbB carrying either a His-tag or a Strep-tag, to the latter of which HRP had been coupled. The ChbB-HRP complexes proved to be considerably more specific for nascent chitin than the previously described WGA-HRP adduct [21] or an assay depending on radioactively labeled substrate [25]. Thus, the newly established test system allows a simultaneous and highly improved scoring of many fractions for the presence of active chitin synthase provoking the synthesis of chitin filaments of either α- or β-type. As a result, the presented test allowed refining the procedure to gain active nano-machineries for chitin biosynthesis (chitosomes). Due to their hydrophobic properties, chitin synthases are difficult to purify to homogeneity [25–27]. Hence, the analysis of their biochemical properties and the identification of accessory compounds will depend on a high quality and quantity of chitosomes. In the yeast *S. cerevisiae*, the chitin synthase CHS3 generates the chitin at the lateral cell wall and at the base of the emerging bud, which is retained at the bud scar of the mother after cell division. Our new finding that 6–8 CHS3-GFP molecules are located within one chitosome suggests their cooperative action to generate nascent chitin. In addition to *S. cerevisiae,* chitosomes have been previously found in the human-pathogen *Candida albicans* and several fungi including *Mucor rouxii*, *N*e*urospora crassa, Blastocladiella emersonii, Phycomyces blakesleeanus*, and, *Agaricus bisporus* (for review, see [13]). In future, it will be interesting to apply our knowledge to fungi, which are of biotechnological importance, or which are pathogens.

In addition, chitin [28] is present within the exoskeleton of different organisms including insects, molluscs, coelenterate and protozoa [29,30]. If the presented strategy will be applied to different organisms producing chitin, the limited current knowledge on the mode of chitin biosynthesis [14,25,26,27,29,30] is expected to multiply considerably.

## Figures and Tables

**Table 1:** Binding ability of the ChbB-HRP conjugate to different polysaccharides.

Polysaccharide	Chitin	Chitosan	Xylan	Cellulose microcrystalline
Binding activity, %	100	13.9	No binding	No binding
